# Binding of 2′,3′-Cyclic Nucleotide Monophosphates to Bacterial Ribosomes Inhibits Translation

**DOI:** 10.1021/acscentsci.2c00681

**Published:** 2022-09-21

**Authors:** Shikha S. Chauhan, Nick J. Marotta, Anna C. Karls, Emily E. Weinert

**Affiliations:** † Department of Biochemistry and Molecular Biology, Penn State University, University Park, Pennsylvania 16802, United States; ‡ Graduate Program in Molecular, Cellular, and Integrative Biosciences, Penn State University, University Park, Pennsylvania 16802, United States; § Department of Microbiology, University of Georgia, Athens, Georgia 30602, United States; ∥ Department of Chemistry, Penn State University, University Park, Pennsylvania 16802, United States

## Abstract

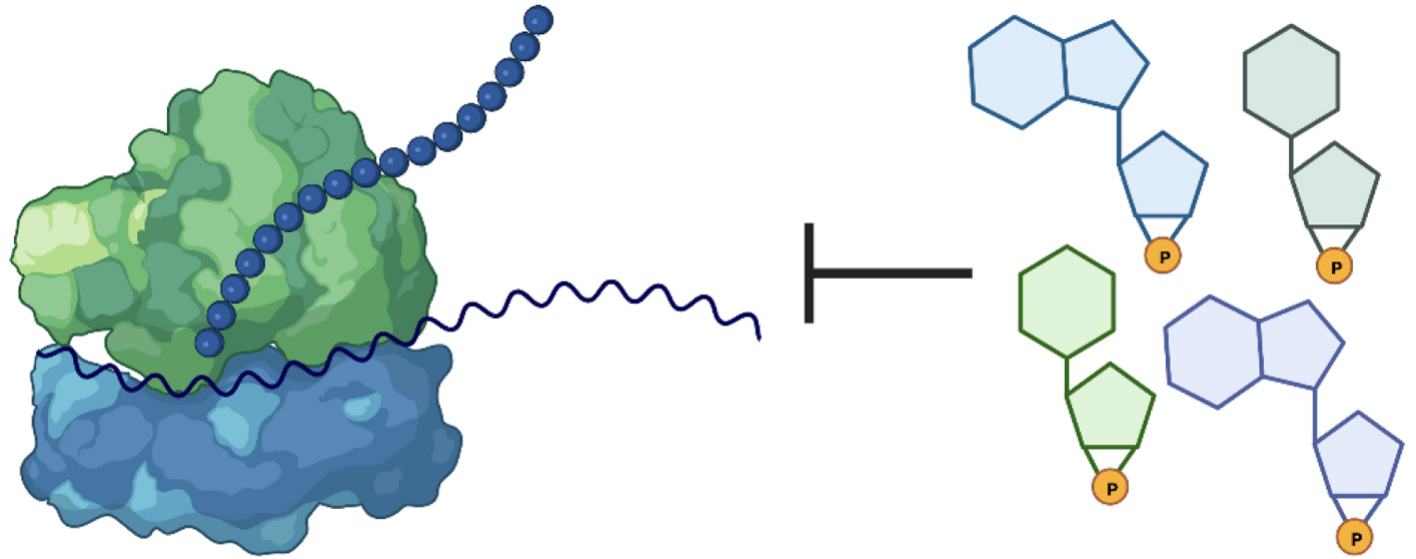

The intracellular small molecules 2′,3′-cyclic nucleotide monophosphates (2′,3′-cNMPs) have recently been rediscovered within both prokaryotes and eukaryotes. Studies in bacteria have demonstrated that 2′,3′-cNMP levels affect bacterial phenotypes, such as biofilm formation, motility, and growth, and modulate expression of numerous genes, suggesting that 2′,3′-cNMP levels are monitored by cells. In this study, 2′,3′-cNMP-linked affinity chromatography resins were used to identify *Escherichia coli* proteins that bind 2′,3′-cNMPs, with the top hits including all of the ribosomal proteins, and to confirm direct binding of purified ribosomes. Using *in vitro* translation assays, we have demonstrated that 2′,3′-cNMPs inhibit translation at concentrations found in amino acid-starved cells. In addition, a genetically encoded tool to increase cellular 2′,3′-cNMP levels was developed and was demonstrated to decrease *E. coli* growth rates. Taken together, this work suggests a mechanism for 2′,3-cNMP levels to modulate bacterial phenotypes by rapidly affecting translation.

## Abstract 2

Cellular small molecules, 2′,3′-cyclic nucleotide monophosphates, bind to bacterial ribosomes, inhibit translation *in vitro*, and modulate *E. coli* growth rates, suggesting a potential mechanism for rapidly altering translation.

## Introduction

Bacteria monitor and respond to their environment using numerous signaling cascades, allowing them to adapt to the environment. These signaling cascades often respond to external stimuli but also are involved in modulating pathways linked to the growth phase and cell density.^1,2^ Many of these cellular processes are regulated by nucleotides that can serve as signaling molecules, such as 3′,5′-cyclic adenosine monophosphate (3′,5′-cAMP) and (p)ppGpp, or as metabolites that act as allosteric regulators, like nucleotide di-/triphosphates (ND/TPs).^3−5^ Nucleotides can modulate processes such as enzymatic activity, transcription, and translation, allowing the cell to adapt to an environmental change or transition into a grow phase. For example, (p)ppGpp is a key regulator of the bacterial stringent response, which is activated under conditions of starvation or environmental stress, and is also involved in regulating the transition from exponential to stationary phase growth.^6−9^

Second messenger signaling can directly alter transcription by affecting binding of transcriptional regulators such as Crp for 3′,5′-cAMP^10^ and DksA for (p)ppGpp.^7^ Similarly, nucleotides second messengers can modulate translation. A number of translation-associated GTPases, such as EF-Tu and IF2, have been demonstrated to bind (p)ppGpp and inhibit translation as a result.^11−14^ The direct inhibition of translation by (p)ppGpp allows for rapid control of protein production when the stringent response is initiated, in addition to the effects caused by changes in cells’ transcriptional profiles.

Another class of cellular nucleotides, termed 2′,3′-cyclic nucleotide monophosphates (2′,3′-cNMPs), has been recently (re)discovered in bacteria and eukaryotes.^15−20^ 2′,3′-cNMPs are products of cellular RNA degradation,^16,21^ and these molecules have been linked to cellular stress in mammals^22−28^ and *Arabidopsis*.^17^ In *E. coli* and *Salmonellaenterica* Typhimurium (*Salmonella* Typhimurium), 2′,3′-cNMPs are upregulated under conditions of amino acid starvation, mRNA decay, ribosome recycling, and alkylating agents.^16,29^ In addition, 2′,3′-cNMP levels have been demonstrated to fluctuate with the growth phase in both *E. coli* and *Salmonella*, suggesting that 2′,3′-cNMPs may be involved in the transition from the exponential to stationary phase. To investigate the role(s) of 2′,3′-cNMPs, our groups have developed tools to modulate 2′,3′-cNMP levels and have demonstrated that decreased 2′,3′-cNMP levels lead to widespread changes in the transcriptomes of *E. coli* and *Salmonella* Typhimurium.^29^

The broad changes in transcription observed when 2′,3′-cNMP levels are decreased suggest that bacteria sense 2′,3′-cNMP levels, likely through binding to cellular proteins. Indeed, recent work in *Arabidopsis* demonstrated that 2′,3′-cAMP is bound by the stress granule and that treatment with 2′,3′-cAMP resulted in increased stress granule production.^20^ Therefore, we generated 2′,3′-cNMP-linked resin to identify 2′,3′-cNMP binding proteins in bacteria. The following studies demonstrate that 2′,3′-cNMPs can bind to bacterial ribosomes, that 2′,3′-cNMP binding inhibits translation *in vitro*, and that increasing intracellular 2′,3′-cNMP levels results in decreased bacterial growth, suggesting that translation can be inhibited by 2′,3′-cNMPs within cells.

## Materials and Methods

### Materials and Reagents

All 2′,3′-cNMPs were purchased from Carbosynth (cat. #’s NA07178, NG145480, NC11429, NU16888). The PurExpress *in vitro* protein synthesis kit (cat. #E6800), DNase I enzyme (cat. #M0303), Thermostable Inorganic Pyrophosphatase (TIPP; cat. #M0296), T7 RNA polymerase (cat. #M0251), RNase inhibitor (cat. #M0314), BSA (cat. #B9000), and nuclease-free water (cat. # B1500L) were purchased from New England Biolabs. The NTP set was purchased from Fischer Scientific. The Epoxy-activated Sepharose 6B, trypsin (cat. #T6567-5x20UG), tris(2-carboxyethyl)phosphine (TCEP (cat. #C4706), iodoacetamide (cat. #I1149), and spermidine (cat. #85558) were purchased from Millipore Sigma. The Bradford assay kit, including BSA, was purchased from Bio-Rad (cat. #5000201). Chemicals were purchased from either Millipore Sigma, Fisher Scientific, or VWR. Bacterial growth media, antibiotics, and buffers were purchased from Research Products Incorporated (RPI), Dot Scientific, or VWR. Plasticware was purchased from VWR.

### Generation of 2′,3′-cNMP-Linked Sepharose

The 2′,3′-cNMP-linked resin was prepared according to the instructions provided in the Epoxy-activated Sepharose 6B (Millipore Sigma cat. # GE17-0480-01) manual. Reaction conditions were optimized for synthesis with 2′,3′-cNMPs. To prepare the resin, 0.3 g of epoxy activated Sepharose 6B was suspended in distilled water to allow it to swell (∼1–2 mL). The resin was then transferred to four fritted columns, and each column was washed with 60 mL of ultrapure water. Separate solutions of 2′,3′-cNMP dissolved in buffer (1 mg/mL of 2′,3′-cNMP in 50 mM phosphate buffer pH 9.5) were generated for each of the four nucleotides (2′,3′-cAMP, 2′,3′-cGMP, 2′,3′-cCMP, 2′,3′-cUMP). Resins were generated with a single linked nucleotide by adding 1 mL of a 2′,3′-cNMP solution to one column containing the swelled resin. The suspension was allowed to rotate end to end at room temperature for 16 h (overnight). The next day, each resin was loaded with one of the four 2′,3′-cNMPs (one column for 2′,3′-cAMP, a second for 2′,3′-cGMP, etc.), loaded into the fritted columns, and allowed to drain. The resin was then washed with 3 mL of coupling buffer. The resin was suspended in 60% glycerol, and the absorbance of a 50% slurry measured at 265 nm confirmed the completion of the reaction. Thin layer chromatography (ethyl acetate: acetonitrile/water/methanol/ammonium hydroxide 6:1.5:1.5:2:0.25 using a silica plate) of the flow-through was performed to determine if the 2′,3′-cNMPs were intact and had not been hydrolyzed to the linear monophosphates. The 2′,3′-cNMP-bound resin was suspended in 1 mL of 10 mM DTT in phosphate buffer (pH 7.5), and the reaction was allowed to shake in an incubator (Southwest Science) at 45 °C overnight to block any remaining epoxy groups. Resins for control experiments were prepared by only activating the epoxy groups with DTT by following the second step as mentioned above. Finally, the resin was washed with alternating buffers: acetate buffer (0.1 M, pH 4) and coupling buffer (pH 8.3) each containing 0.5 M NaCl to remove any excess of uncoupled 2′,3′-cNMPs. The final resins were stored in coupling buffer at 4 °C.

### *E. coli* and *Salmonella* Typhimurium Cell Lysate Preparation

*E. coli* BW25113 *ΔcpdB* (Keio Collection) was streaked onto LB agar containing kanamycin (50 μg/mL) and incubated overnight at 37 °C. Isolated colonies were picked and used to inoculate 10 mL of M9 media + kanamycin (50 mg/mL; M9+Kan) and then incubated at 37 °C with 220 rpm shaking overnight. The resulting starter culture then was inoculated 1:100 into 100 mL of M9+Kan in a sterile 250 mL Erlenmeyer flask and incubated with shaking at 220 rpm, 37 °C until the OD_600_ reached 0.5–0.6. Cells were harvested by centrifugation at 4000 rpm, 4 °C for 20 min in an Allegra X-30 R centrifuge (Beckman), and the resultant cell pellets were frozen at −80 °C until further use. Lysates were prepared by thawing the frozen cell pellets on ice, resuspending in 10 mL of TRIS buffer (50 mM, pH 7.5) and then lysed by sonication (QSonica). Cellular debris from the lysed cells was removed by centrifugation at 10000*g* at 4 °C for 20 min, and the supernatant was collected for pull-down assays.

Isolated colonies of *S*. *enterica* subspecies *enterica* serovar Typhimurium strain 14028s (ATCC; *S.* Typhimurium) from streaks on LB agar were used to inoculate 5 mL of LB for overnight cultures; 1 mL of overnight cultures was used to inoculate 100 mL of M9-glucose minimal medium, and cultures were processed as described above for *E. coli*.

### 2′,3′-cNMP Binding Protein Pull-Down

Before starting the pull-down experiment, 200 μL of 2′,3′-cNMP-linked Sepharose resin (stored in phosphate coupling buffer) was first washed with 50 mM TRIS buffer pH 7.5. Epoxy-Sepharose that had been blocked with DTT was used as a control and prepared in an analogous manner. The resin was then suspended in 500 μL of *E. coli* BW25113 *ΔcpdB* or *S*. Typhimurium 14028s lysate and rotated on an end-to-end rotator at 4 °C for 3–4 h. The resin was then loaded in a small gravity column to perform the chromatography. The columns were first washed with 1 mL of wash buffer (50 mM TRIS buffer, pH 7.5, 20 mM NaCl). The washing was then followed by elution with 400 μL of elution buffer containing 50 mM, 200 mM, 500 mM, and 1 M NaCl in 50 mM TRIS buffer, pH 7.5. Fractions eluted with each concentration of salt were collected. Finally, the beads were heated at 56 °C for 10 min in 50 mM TRIS buffer, pH 7.5, 1 M NaCl, and the supernatant was collected to ensure that all of the bound protein was eluted. The samples were submitted for quantitative mass spectrometry for proteomics.

### In Solution Protein Digestion

All solutions were prepared in a digestion buffer consisting of 16 mg/mL ammonium bicarbonate in water. The reducing agent was 30 mg/mL tris(2-carboxyethyl)phosphine (TCEP), and the alkylating agent was 18 mg/mL iodoacetamide, both freshly prepared in the digestion buffer. A stock solution of trypsin was prepared by mixing 20 μg of proteomics grade trypsin in 20 μL of 50 mM acetic acid, pH ≈ 3, aliquoted and stored at −20 °C. To prepare activated (or working) trypsin solution, trypsin stock solution (Thermo Scientific cat. #1862748) was diluted with digestion buffer 10-fold to a concentration of 0.1 μg/μL.

Before the digestion was started, buffer exchange was performed for desalting the protein samples using a 3,000 MWCO centrifugal filter. The samples were centrifuged at 14000 rpm for 5 min using 50 mM, pH 7.5 TRIS buffer, and the process was repeated three times for complete desalting. To begin digestion, 15 μL of digestion buffer was combined with 3 μL of TCEP and 12 μL of sample solution containing 0.025 μg to 10 μg protein (total volume 30 μL). The mixture was denatured/reduced at 60 °C for 10 min, cooled down to room temperature. Next, 3 μL of the alkylating agent was added, and the mixture was incubated in the dark at room temperature for 20 min. Finally, 5 μL of fresh activated trypsin was added to the samples and incubated at 37 °C overnight.

### Mass Spectrometry (Proteomics) of *E. coli*

#### Nano-LC MS^2^

The samples were reconstituted in 10 μL of solvent A (100% water and 0.1% formic acid), and 2 μL was injected on to a Waters column (25 cm, 1.7 μm, 130 Å) preceded in-line by a Waters nanoEase M/Z symmetry C18Trap column (100 Å, 5 μm, 10 μM × 20 mm) heated to 40 °C. A Waters Acquity UPLC M Class Nano LC system was used to deliver the following gradient at 300 nL/min into a Sciex 5600 TripleTOF: 0–35% linear gradient of mobile phase B (100% aqueous acetonitrile containing 0.1% formic acid) in mobile phase A (0.1% formic acid in 100% water) over 25 min, followed by a 5 min isocratic stage at 80% B, and a 10 min isocratic wash at 0% B to re-equilibrate the column. The following settings were used for the Sciex 5600 TripleTOF: parent scans were acquired for 250 ms, and then up to 50 MS/MS spectra were acquired over 2.5 s for a total cycle time of 2.8 s.

#### LC MS^2^ Data Analysis

The MS/MS spectra were searched against a concatenated protein sequence database containing the NCBI RefSeq database for *E. coli* K12 (Ecoli_K12; 4518 protein sequences) plus a database containing the protein sequences for 536 common lab contaminants (compiled in the Penn State College of Medicine Mass Spectrometry and Proteomics Facility (RRID:SCR_017831)), for a total of 5054 forward protein sequences, plus a decoy database containing the reversed sequences of those 5054 forward protein sequences. False positive “hits” from that reversed database were used to calculate the False Discovery Rate, using ProteinPilot 5.01 (Sciex) with the Paragon algorithm.^30^ The precursor and fragment tolerances were set to 10 ppm, dynamic modifications included Oxidation (+15.995 Da, M) and Deamidation (+0.984 Da, N, Q), and static modification was Carbamidomethyl (+57.021 Da, C).

### Mass Spectrometry (Proteomics) of *Salmonella* Typhimurium

#### Nano-LC MS^2^

The tryptic peptides were dissolved in 10 μL of 4% acetonitrile, 0.1% formic acid, and 1.5 μL was loaded and separated on an Acclaim PepMap RSLC column (75 μm × 25 cm, C18, 2 μm, 100 Å, Thermo) with a 50 min 5–35% linear gradient of mobile phase B (80% aqueous acetonitrile containing 0.1% formic acid) in mobile phase A (0.1% formic acid in water), followed by a 10 min isocratic 95% B. The gradient was delivered by an Easy-nLC 1200 system (Thermo) at 300 nL/min. An Orbitrap Eclipse mass spectrometer (Thermo Scientific) data acquisition method was based on the “Single Cell LFQ” template with the following modifications: cycle time 2 s, maximum injection time for MS2 250 ms, charge states 2–6.

#### LC MS^2^ Data Analysis

The mass spectra were processed using Proteome Discoverer 2.5 (Thermo). The proteins were identified by searching the data against *Salmonellatyphimurium* database downloaded (UP000002695) from UniprotKB on 06/25/2021 and a common contaminants database (Thermo). The precursor and fragment tolerances were set to 10 ppm, dynamic modifications included Oxidation (+15.995 Da, M) and Deamidation (+0.984 Da, N, Q), and static modification was Carbamidomethyl (+ 57.021 Da, C).

### *In Vitro* Transcription

All steps were conducted in nuclease-free conditions. A 5× transcription buffer stock was prepared by mixing the following: 250 mL of 1 M Hepes pH 7.5, 100 mL of 1 M DTT, 50 mL of 1 M MgCl_2_, 10 mL of 0.5 M spermidine, 25 mL of 100 mg/mL BSA, and 65 mL of RNase-free H_2_O and mixing until clear. For a 100 μL scale transcription reaction, the following were added to the tube on ice: 20 μL of 5× transcription buffer, 1 μL of 1 M DTT, 2.7 μL of 1 M MgCl_2_, 30 μL of 100 mM NTP mix, 4 μL of NanoLuc luciferase DNA template,^31^ 0.5 μL of TIPP, 0.2 μL of RNase inhibitor, 5 μL of T7 RNA polymerase, and 36.6 μL of nuclease-free H_2_O. The tube containing the transcription reaction was then incubated at 37 °C for 3 h, followed by treatment with DNase I. DNase I treatment was performed by removing 10 μL of reaction mix, followed by addition of 10 μL of DNase I reaction buffer (RNase free) and 2 units of RNase free DNase I. The reaction contents were mixed and incubated at 37 °C for 10 min, and then the reaction was terminated by adding 5 mM (final concentration) EDTA (1 μL of 0.5 M EDTA-HCl salt) and heating the reaction at 75 °C for 10 min. The *in vitro* prepared mRNA was used for *in vitro* protein synthesis reactions.

### *In Vitro* Protein Synthesis

The PurExpress *in vitro* protein synthesis kit was utilized for both coupled transcription-translation and translation assays. The reaction mixture was prepared in a microcentrifuge tube by adding 2 μL of solution A, 1.5 μL of solution B, 1 μL of NanoLuc DNA template^31^ or mRNA (final ∼15–20 ng/μL), and 3.5 μL of either nuclease-free H_2_O (control experiments), 2′,3′-cNMP solution (in nuclease-free water), 2′(3′)-GMP solution (in nuclease free water; Millipore Sigma cat. #G8002), or cyclic dimeric guanosine monophosphate (c-di-GMP; in nuclease free water; Cayman Chemical Company cat. #CAYM-17144-1). The samples were then incubated at 37 °C for 3 h. After incubation, sample tubes were moved to room temperature and allowed to sit for ∼5 min. In preparation for activity assays, Nano-Luc substrate buffer was thawed at room temperature, and a substrate solution was prepared by adding 1 μL of substrate (Furimazine; Promega N1110) to 50 μL of substrate buffer from Promega. To assess levels of synthesized Nano-Luc enzyme, 8 μL of the prepared substrate solution was transferred to each tube of the protein synthesis reaction and mixed well. Ten microliters of each protein synthesis-substrate mixture was transferred to a well of a 96-well microplate (Costar white plate; VWR), and the luminescence produced by Nano-Luc enzyme in the reactions was quantified by measuring total light production (luminescence) using Cytation 5 plate reader (Agilent/Biotek).

### Ribosome-2′,3′-cGMP Binding Experiment

100 μL of 2′,3′-cGMP linked Sepharose resin was suspended in 66 μL (2 mg, 13.3 μM) of *E. coli* cell 70S ribosome (New England BioLabs cat. #P0763; supplied in 20 mM HEPES-KOH pH 7.6, 10 mM Mg(OAc)_2_, 30 mM KCl, and 7 mM β-mercaptoethanol) and rotated on an end-to-end rotator at 4 °C for 3 h. The resin was then loaded in a small gravity column to perform the chromatography. The columns were first washed (3 column volumes) with wash buffer (20 mM HEPES-KOH pH 7.6, 10 mM Mg(OAc)_2_, 30 mM KCl, and 7 mM β-mercaptoethanol). The washing was then followed by elution (2 column volumes) with elution buffer containing different concentrations of 2′,3′-cGMP in the wash buffer. Similarly, a control experiment was set up but instead of eluting with a different concentration of 2′,3′-cGMP, the control column was washed only with buffer to calculate the difference in the level of eluted ribosome. All the samples were collected, and the ribosome concentration was checked by quantifying the absorbance at 595 nm using a Bradford test based on bovine serum albumin. Samples were quantified in a 96-well plate, and the absorbance was measured using an Epoch 2 plate reader (Agilent/Biotek).

### Increased 2′,3′-cNMP Production

Gene inserts were synthesized by Genscript (Piscataway, New Jersey, U.S.) and cloned into a pET31b+ plasmid between the *Bgl*III and XhoI restriction sites. Restriction site sequences are in italics, and T7/mutated T7 binding sites are underlined (Table 1).

**Table 1:** Gene Sequences for the pNoRBS and pNoT7 Constructs Used to Increase 2′,3′-cNMP Levels and As a Control, Respectively

construct	sequence
pNoRBS	*AGATCT*TAATACGACTCACTATAGGACTAGTCGTCGGCGAAGGACGGGTCCAGCGTTCGCGCTGTTGAGTAGAGTGTGAGCGCCCTCGTACAGCCCTAGCATAACCCCTTGGGGCCTCTAAACGGGTCTTGAGGGGTTTTTTG*CTCGAG*
pNoT7	*AGATCT*AGGCCTAGTCCACCGCGGGACTAGTCGTCGGCGAAGGACGGGTCCAGCGTTCGCGCTGTTGAGTAGAGTGTGAGCGCCCTCGTACAGCCCTAGCATAACCCCTTGGGGCCTCTAAACGGGTCTTGAGGGGTTTTTTG*CTCGAG*

The plasmids were transformed into Novagen Tuner (DE3) (*ompT hsdSB (rB– mB−) gal dcm lacY1*) competent cells (Sigma, Cat: 70623-3) or *Escherichia coli* BW25113 (DE3) (*lacI*^q^*rrnB*_T14_ Δ*lacZ*_WJ16_*hsdR514* Δ*araBAD*_AH33_ Δ*rhaBAD*_LD78_) cells that were made competent using RbCl chemical competency.

### Quantification of Cellular 2′,3′-cNMP Levels

Bacterial growth and 2′,3′-cNMP isolation was performed as previously described, with minor modifications.^16,29^ Isolated colonies were grown overnight in 2 mL of M9 minimal media (supplemented with 0.2% casamino acids, 0.4% glucose) and inoculated 1:100 into fresh media containing 100 μM IPTG and, if necessary, 100 μg/mL ampicillin (DOT Scientific). Growths occurred in 50 mL VWR centrifuge tubes (sterile, polypropylene, with caps loose for aeration; Cat: 21008-242), under 37 °C incubation and 225 rpm shaking. When cells had grown for 16 h, the absorbance of each culture was measured at 600 nm, and the cells were harvested by centrifugation at 3000*g* and 4 °C for 30 min. The supernatant was aspirated and reserved for absorbance measurement, and then the pellets were frozen in liquid nitrogen and stored at −80 °C until extraction. All data represent at least three biological replicates.

To extract 2′,3′-cNMPs, 1 mL of a 2:2:1 acetonitrile/methanol/water mixture was added to the still frozen pellets, allowed to thaw on ice, and resuspended with a pipettor. The resuspension was transferred to 2 mL microcentrifuge tubes and sonicated in icy water in a cup horn (Qsonica Q500 and part #431C2) at an amplitude of 70% for 3 min in 30 s on/off steps (6 min total). Samples were then centrifuged at 4 °C and 3500*g* for 10 min. Supernatant was transferred to new tubes and concentrated to dryness at room temperature using an Eppendorf Vacufuge vacuum centrifuge. The extracts were stored at −80 °C until used for LC:MS/MS. Extracts were resuspended with 250 μL of Molecular Biology grade water (VWR,) containing 0.5 μM 8-bromoadenosine 3′,5′-cyclic monophosphate (8-Br-cAMP; Millipore Sigma cat. #203800) as an internal standard. Authentic standards of 2′,3′-cNMPs were obtained as monosodium salts and prepared at concentrations of 10 μM, 1 μM, 0.1 μM, and 0.01 μM each in a 0.5 μM 8-Br cAMP solution. 2′,3′-cAMP, 2′,3′ cGMP, 2′,3′ cCMP, and 2′,3′ cUMP were obtained from Carbosyth. Concentrations of nucleotide stock solutions were verified by UV–vis spectrometry (Cary Series, Agilent Technology) using Beer’s Law and extinction coefficients from 5′-NMPs.^32^

Ten microliters of sample were separated by reverse phase HPLC using a Prominence 20 UFLCXR system (Shimadzu,) with a Waters BEH C18 column (100 mm × 2.1 mm 1.7 μM particle size) maintained at 55 °C and a 20 min aqueous acetonitrile gradient at a flow rate of 250 μL/min. Solvent A was HPLC grade water with 0.1% formic acid and Solvent B was HPLC grade acetonitrile with 0.1% formic acid. The initial condition were 0% A and 100% B, increasing to 2% B at 5 min, 10% B at 10 min, and 75% B at 16 min where it was held for 0.1 min before returning to the initial conditions. The eluate was delivered into a 5600 (QTOF) TripleTOF using a Duospray ion source (AB Sciex,). The capillary voltage was set at 5.5 kV in positive ion mode, with a declustering potential of 50 V. The mass spectrometer was operated with a 100 ms TOF scan from 100 to 1000 *m*/*z*, and 7 MS/MS 100 ms product ion scans (parent ion 408.0, 410.0, 330.1, 331.1, 346.1, 306.1 and 307.1) per duty cycle using a collision energy of 25 V with a 5 V spread.

Data were processed using PeakView version 2.1.0.11041. Concentrations were quantified using an internal standard method, with 8-Br 3′,5′-cAMP serving as the internal standard. To create a standard curve of comparing peak area to nucleotide concentration, the area of the nucleotide standard peak was divided by the area of the internal standard peak and plotted against the quotient of nucleotide standard concentration over the internal standard concentration. A linear regression model was used to generate the standard curves, and the response within standards remained linear. 2′,3′-cNMPs were adjusted based on extraction recovery values determined previously^33^ and normalized to cell density. Significance was determined by two sided students *t* tests, or ANOVA, with a *P*-value <0.05 considered to be significant.

### Growth Curves for Cells with Increased 2′,3′-cNMP Levels

Wild type Tuner cells, Tuner pNoRBS, and Tuner pNoT7 were streaked onto LB agar containing 100 μg/mL ampicillin where appropriate and incubated overnight at 37 °C. Colonies were picked from the plates and used to inoculate 2 mL of M9 minimal media supplemented with 0.4% glucose and 0.2% casamino acids in 15 mL culture tubes containing 100 μg/mL ampicillin where appropriate. The cultures were incubated at 37 °C and 250 rpm overnight.

A clear, sterile 24-well plate (VWR) was used for the growths. Two milliliters of M9 minimal media supplemented with 0.4% glucose and 0.2% casamino acids, 100 μM IPTG, and (if necessary) 100 μg/mL ampicillin were added to each well that would be used. The media was aliquoted from a larger mixture to ensure that all samples had the same concentration of IPTG. Twenty microliters of the appropriate overnight culture was added to each well. The plate was covered with a BreatheEasy film to avoid evaporation while allowing for oxygen diffusion. The plate was incubated at 37 °C and 160 rpm double orbital shaking for 24 h in an Epoch 2 microplate spectrophotometer (Agilent/Biotek) with sampling every 30 min.

## Results and Discussion

### 2′,3′-cNMPs Bind Ribosomes

The observation of significant dysregulation of transcript levels (>500 transcripts in *E. coli* and ∼170 transcripts in *Salmonella* Typhimurium) in strains expressing a 2′,3′-cyclic nucleotide phosphodiesterase versus strains expressing an inactive phosphodiesterase variant led us to investigate the proteins and pathways involved in sensing and responding to 2′,3′-cNMPs.^16,29^ Given the lack of information on protein domains that bind 2′,3′-cNMPs, we utilized an untargeted approach to identify molecules that interact with 2′,3′-cNMPs in *E. coli.* To do so, we generated 2′,3′-cNMP-linked resins by coupling each 2′,3′-cNMP (A/U/G/C) to epoxy-activated Sepharose beads. We chose to use epoxy-linked resin because multiple nucleophilic sites on each nucleotide (5′-OH, nitrogen within nucleotide base) could potentially react with the epoxide and did not rely on any knowledge of preferred 2′,3′-cNMP binding orientation. Nucleotide-bound and control beads were used in parallel for affinity purification of proteins from *E. coli* BW25113, followed by subsequent trypsin digestion and mass-spectrometry analysis. The *E. coli* BW25113 Δ*cpdB* strain was used for the pull-down experiments to preserve the 2′,3′-cNMP-linked resin, as CpdB is known to exhibit 2′,3′-cyclic phosphate hydrolysis activity.^34−36^ Overall, the mass spectrometry identified over 100 proteins from pull-downs performed with all four resins, many of which were identified multiple times from columns with different nucleotide bases. Every ribosomal protein was identified, with most ribosomal proteins being identified in samples eluted from multiple/all nucleotide columns (Table S1; Data set S1). These results suggested that 2′,3′-cNMPs might bind to the ribosome and therefore could play a role in modulating translation.

**Figure 1:**
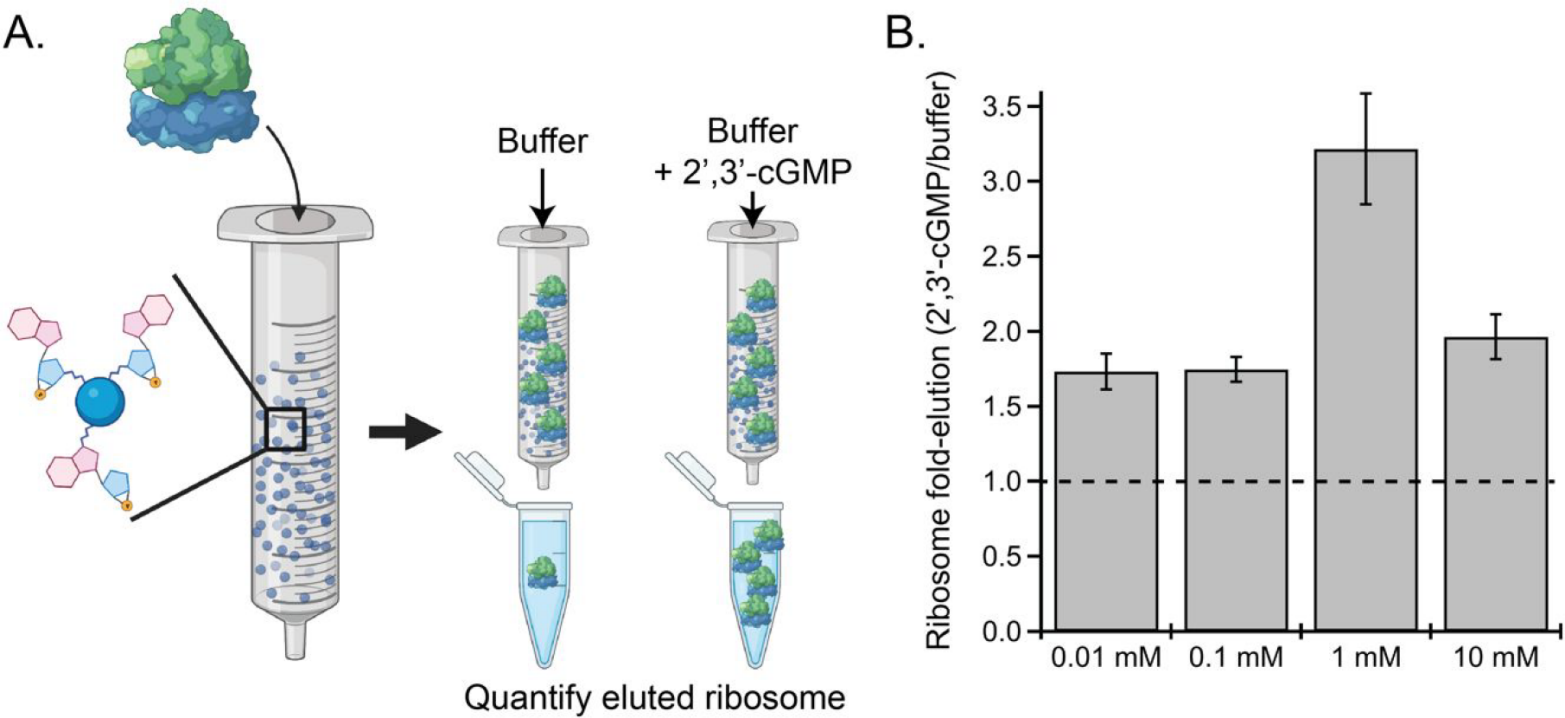
Ribosome binding to 2′,3′-cGMP-linked resin. (A) Schematic of experimental design. Ribosome is loaded into columns of 2′,3′-cGMP resin; one column serves as a control and is only washed with buffer, while the second column is washed with buffer containing 2′,3′-cGMP. Eluted ribosome is quantified in each fraction. (B) Quantification of ribosome eluted from 2′,3′-cGMP resin. The dashed line represents an equal amount of ribosome eluted in the presence of buffer + 2′,3′-cGMP and buffer. 2′,3′-cGMP competes with 2′,3′-cGMP for binding to the ribosome, resulting in greater elution.

To validate the proteomics data, we sought to determine if ribosome binding to the 2′,3′-cNMP-linked resins was specific for 2′,3′-cNMPs or due to nonspecific interactions. Purified *E. coli* 70S ribosomes were incubated with 2′,3′-cGMP-linked Sepharose resin; 2′,3′-cGMP-linked resin was chosen because all ribosomal proteins were identified from those pull-down samples. The columns were then washed with either buffer containing increasing amounts of 2′,3′-cGMP or with buffer (without 2′,3′-cGMP) as a control to determine if ribosomes could be specifically eluted with 2′,3′-cNMPs. Our results indicate that the inclusion of 2′,3′-cGMP in the elution buffer resulted in up to 3-fold greater *E. coli* ribosome elution, with a maximum fold increase in ribosome eluted at 1 mM 2′,3′-cGMP (Figure 1). These studies confirm that the *E. coli* 70S can bind to and be eluted by 2′,3′-cNMPs.

To determine if 2′,3′-cNMP-ribosome interactions are unique to *E. coli* or might be found more broadly in bacteria, pull-downs using 2′,3′-cNMP-linked resin were performed in an additional species. *Salmonella* Typhimurium was chosen for the pull-down studies because it has been demonstrated to produce 2′,3′-cNMPs and exhibit 2′,3′-cNMP-dependent differences in transcription. When pull-downs were performed in *S.* Typhimurium, we identified 34 ribosomal proteins by mass spectrometry (Table S2, Data set S2). These results demonstrate that both *E. coli* and *S.* Typhimurium ribosomes interact with 2′,3′-cNMPs, suggesting a role for 2′,3′-cNMPs in translational regulation in the γ-proteobacteria.

### 2′,3′-cNMPs Inhibit *in Vitro* Translation

**Figure 2:**
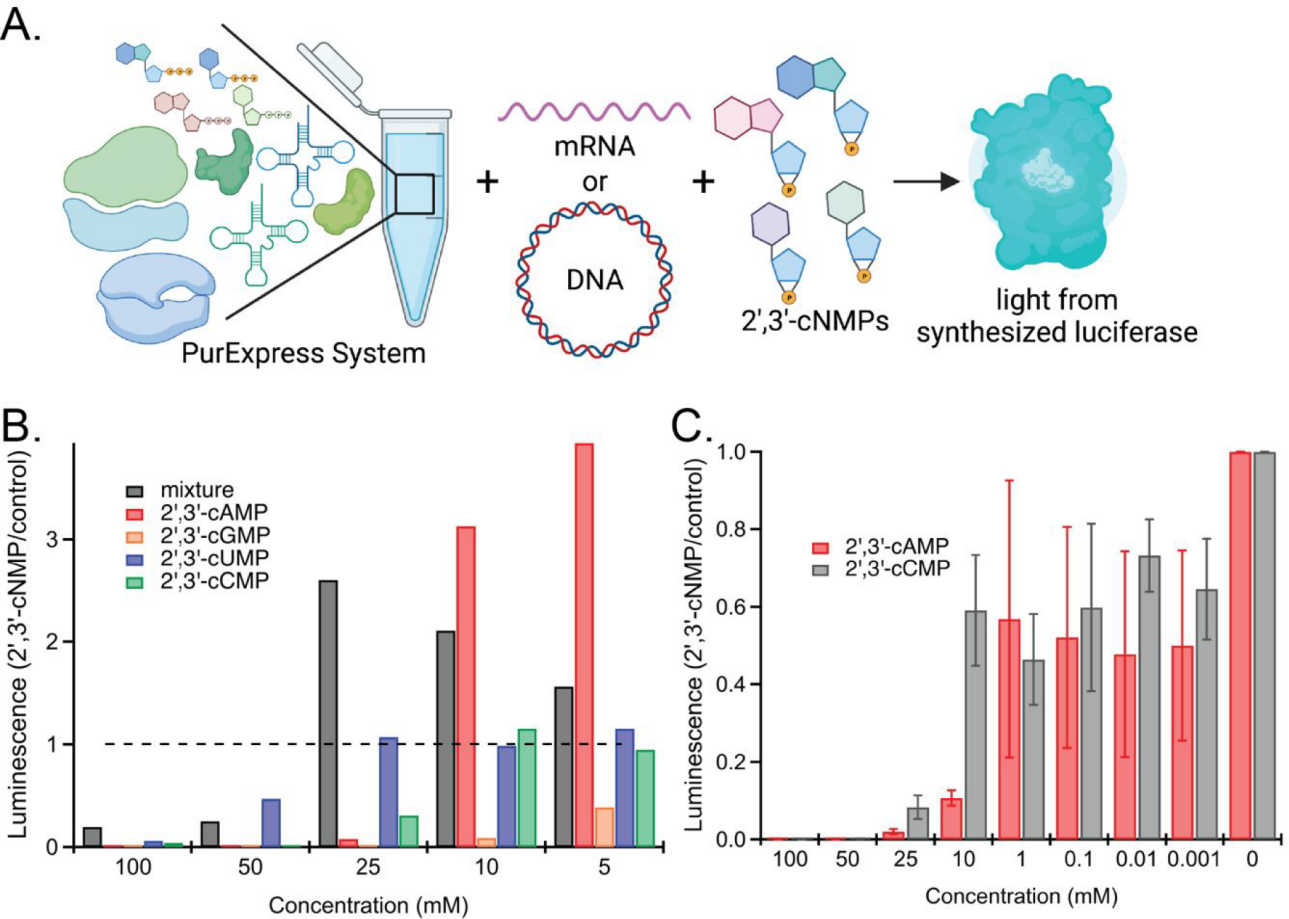
*In vitro* translation assays. (A) Schematic of *in vitro* transcription/translation and translation assays using the PurExpress kit, a NanoLuc reporter, and varying levels of 2′,3′-cNMPs. (B) Addition of 2′,3′-cNMPs inhibits production of NanoLuc in a coupled transcription/translation assay, but high levels of 2′,3′-cAMP result in greater luminescence. (C) 2′,3′-cNMPs inhibit NanoLuc production in an *in vitro* translation assay (mRNA transcribed separately). Error bars represent standard deviation. Levels of inhibition are significant, relative to control (0 mM), for all concentrations except 1 mM 2′,3′-cAMP and 100 μM 2′,3′-cCMP. 2′,3′-cAMP: 10–100 mM, *P* < 0.0001; 100 μM, *P* < 0.0017; 10 M, *P* < 0.031; 1 M, *P* < 0.028. 2′,3′-cCMP: 25–100 mM, *P* < 0.0001; 10 mM, *P* < 0.0066; 1 mM, *P* < 0.0087; 1–10 μM, *P* < 0.035.

We next examined the effects of 2′,3′-cNMPs on translation by performing *in vitro* translation assays^37^ in the presence/absence of 2′,3′-cNMPs (Figure 2). NanoLuc luciferase was chosen as a reporter gene due to its enhanced stability, smaller size, >150-fold increase in luminescence, and previous usage in *in vitro* translation assays.^31,38^ Initially, coupled transcription-translation assays were performed using the PurExpress *in vitro* assay kit and high (5–100 mM) concentrations of 2′,3′-cNMPs to determine if there was any effect on translation. A mixture of the four 2′,3′-cNMPs was included in the coupled transcription-translation assays to mimic the occurrence of all four 2′,3′-cNMP in the cell. We observed that high levels of 2′,3′-cNMPs (50 and 100 mM) inhibited luciferase production; however, lower concentrations of 2′,3′-cNMPs did not inhibit the *in vitro* transcription/translation reactions but resulted in increased luminescence instead (Figure S1). To investigate how different 2′,3′-cNMPs effect the inhibition, we measured coupled transcription-translation (via NanoLuc chemiluminescence) in the presence of individual 2′,3′-cNMPs. Although both 2′,3′-cAMP and 2′,3′-cGMP showed more inhibition than 2′,3′-cUMP at high concentrations (50 and 100 mM; >90% vs 0–50%, respectively), the addition of lower concentrations of 2′,3′-cAMP (5 mM and 10 mM) resulted in higher luminescence (∼3-fold) than the control (Figure 2B). These results suggest that 2′,3′-cAMP might differentially effect transcription versus translation, resulting in the concentration-dependent effects on NanoLuc production.

To delineate the inhibitory effects of 2′,3′-cNMPs on transcription versus translation, we performed *in vitro* protein synthesis using NanoLuc mRNA, instead of coupled transcription/translation from a DNA template (Figure 2C). Reactions were performed with one purine (2′,3′-cAMP) and one pyrimidine (2′,3′-cCMP) to directly compare their effects on translation. Overall, 2′,3′-cAMP exhibited greater inhibition of translation as compared to 2′,3′-cCMP. While 2′,3′-cAMP showed ∼90% inhibition up to 10 mM and ∼50% inhibition at lower concentrations, 2′,3′-cCMP showed ∼90% inhibition only up to 25 mM. In addition, greater inhibition was observed in translation reactions than coupled transcription-translation reactions, even with the same concentration of 2′,3′-cNMPs.

Unlike in the coupled transcription/translation assays, no enhanced luminescence relative to the control was observed at low 2′,3′-cAMP concentrations. 2′,3′-cAMP exhibited greater inhibition of translation as compared to 2′,3′-cCMP at the 10 mM concentration (90% vs 40% reduction, respectively), but both 2′,3′-cNMPs showed significant inhibition of translation at 25, 50, and 100 mM concentrations, with >90% reduction in luminescence. In addition, greater inhibition was observed in translation reactions than coupled transcription-translation reactions at concentrations below 10 mM, which are those within the physiologically relevant range.^16^ These results suggest that cells can modulate protein synthesis by altering cytoplasmic 2′,3′-cNMP levels. The fluctuations in 2′,3-cNMP concentration (ranging from <500 fM from >100 μM) over the growth phases^16,29^ may help to modulate translation as the cells alter behavior and physiological state. Similarly, the increase in 2′,3′-cNMP levels upon amino acid starvation^16^ may allow the cells to rapidly inhibit translation and preserving amino acid stores.

Given the prevalence of positively charged residues within ribosomal proteins and known nucleotide binding sites on various translation related proteins, we sought to determine if the effects of 2′,3′-cNMPs on translation were due to nonspecific interactions. Since the purine-containing 2′,3′-cNMPs exhibited similar inhibitory effect *in vitro*, the effects of a linear analogue of 2′,3′-cGMP (2′(3′)-GMP (mixed regioisomer)) and cyclic dimeric guanosine monophosphate (c-di-GMP, a bacterial second messenger)^1^ were investigated using the *in vitro* translation assay. 2′(3′)-GMP was chosen to test if the 2′,3′-cyclic phosphate was required for inhibition, while c-di-GMP was chosen to determine if a purine-containing cyclic-dinucleotide could inhibit translation. Neither 2′(3′)-GMP nor c-di-GMP exhibited statistically significant inhibition of translation at 1 mM and 10 mM, concentrations at which 2′,3′-cAMP exhibits ∼40% and ∼90% inhibition, respectively. These data suggest that the 2′,3′-cyclic nucleotide structure is required for inhibiting translation, potentially through specific binding contacts on the ribosome.

### 2′,3′-cNMPs Alter Bacterial Growth

The effect of 2′,3′-cNMP levels on translation in cells was investigated by using an inducible plasmid containing a short, noncoding RNA gene to increase cellular 2′,3′-cNMP levels. The abundance of untranslatable RNA in *E. coli* cells was expected to result in increased RNA degradation, leading to more substrates for RNase I to degrade into 2′,3′-cNMPs. Induction of transcription of the noncoding RNA resulted in a statistically significant ∼6-fold increase in 2′,3′-cAMP and 2′,3′-cGMP concentrations in the stationary phase, when compared to wild type (Figure S3). This effect size is similar to the change in 2′,3′-cNMP abundance found between the early and late stationary phase in wild type cells. The negative control-plasmid strain, containing the same plasmid with a mutated T7 promoter to decrease transcription, yielded an ∼2 fold increase in 2′,3′-cAMP and GMP levels, relative to wild type cells, when induced with IPTG under the same conditions. This increase in concentration observed in the control is due to the maintenance of the plasmid and its antibiotic resistance gene, which also generates RNA, and/or leaky expression despite the mutated promoter. Nonetheless, a highly statistically significant (*P* < 10^–6^) ∼3 fold increase in 2′,3′-cAMP and 2′,3′-cGMP levels in the test strain relative to the negative control-plasmid strain demonstrates that increased RNA abundance leads to a significant change in 2′,3′-cNMP concentration at an order of magnitude similar to that observed in physiological responses such as to amino acid starvation or changes in growth phase.

**Figure 3:**
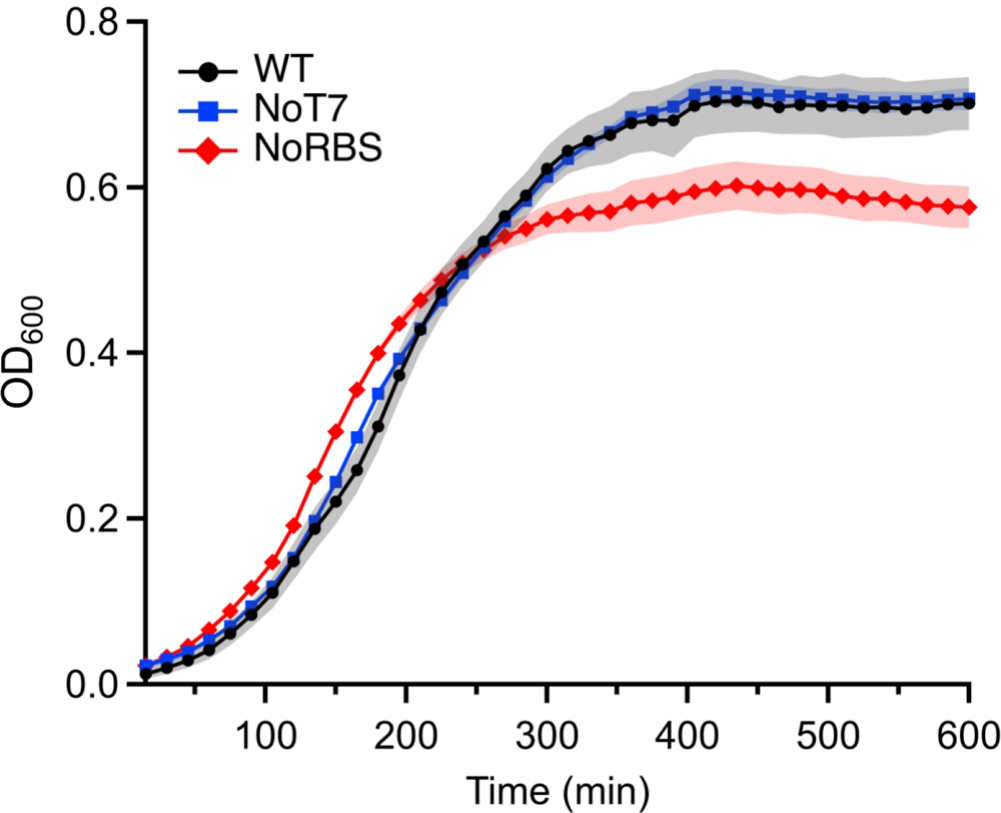
Growth curves of *E. coli* BL21 Tuner (DE3) wild type (WT) cells, cells with an ∼2-fold increase in 2′,3′-cNMP levels (WT cells with a plasmid that has a heavily mutated T7 promoter and no ribosome binding site; NoT7) and WT cells with an ∼5–7-fold increase in 2′,3′-cNMP levels (WT cells with a plasmid that has no ribosome binding site; NoRBS).

Induction of 2′,3′-cNMP production resulted in decreased maximum cell density reached in the stationary phase (∼0.72 vs 0.53 for WTand pNoRBS, respectively; Figure 3). However, the amount of time required to reach half maximal OD_600_ is shorter for the pNoRBS-containing strain vs WT and the pNoT7 containing strain (148 min vs ∼185 min, respectively), and the growth rate is faster (38 min^–1^ vs ∼56 min^–1^, respectively; Figure S4). Taken together, the growth curves suggest that increased 2′,3′-cNMP levels result in faster strain growth during the exponential phase but that shifting to the stationary phase more quickly is accompanied by decreased cell density.

As 2′,3′-cNMP levels fluctuate with the growth phase in both *E. coli* and *S. typhimurium*,^16,29^ with higher levels in the exponential phase, increasing the 2′,3′-cNMP concentration *in vivo* may modulate growth-phase specific translation. The decreased cell density in the stationary phase may be due to increased levels of 2′,3′-cNMPs inhibiting translation, as was observed for the *in vitro* translation inhibition results.

## Conclusions

Our results have identified a previously uncharacterized interaction between 2′,3′-cNMPs and bacterial ribosomes, which inhibits translation *in vitro*. Increasing 2′,3′-cNMP levels in cells results in altered growth rates and stationary phase cell density, suggesting that bacteria can sense 2′,3′-cNMP concentrations to modulate cellular physiology. The effects on cell growth are likely due to inhibition of translation but may also be due to interactions of 2′,3′-cNMPs with other cellular proteins. These studies provide the foundation for identifying 2′,3′-cNMP binding site(s) on the ribosome and potentially could be developed into new translation inhibitors. The present study builds a platform to explore the binding of ribosomes with the 2′,3′-cNMPs and therefore is an important discovery for the development of antibiotics.

## Supporting Information Available

Additional figures, tables (PDF)
Data sets of all identified proteins (XLSX-1, XLSX-2)
