# Supplementary material for: Binding of 2′,3′-Cyclic Nucleotide Monophosphates to Bacterial Ribosomes Inhibits Translation
Source: ACS Cent Sci. 2022 Sep 21;8(11):1518–26. doi: 10.1021/acscentsci.2c00681 (PMC9686202; doi:10.1021/acscentsci.2c00681)
Supplement: Supplementary file 1 — oc2c00681_si_001.pdf [file oc2c00681_si_001.pdf]

Supporting Information

Binding of 2',3'-cyclic nucleotide monophosphates to bacterial ribosomes inhibits translation

Shikha S. Chauhan[1], Nick J. Marotta[2], Anna C. Karls[3], and Emily E. Weinert[1,4]*

[1] Department of Biochemistry and Molecular Biology, Penn State University, University Park, PA, 16803, USA
[2] Graduate Program in Molecular, Cellular, and Integrative Biosciences, Penn State University, University Park, PA, 16803, USA
[3] Department of Microbiology, University of Georgia, Athens, GA, 30602, USA
[4] Department of Chemistry, Penn State University, University Park, PA, 16803, USA
* To whom correspondence should be addressed. Tel: +1 814-865-3719; Email: emily.weinert@psu.edu

Table S1: *E. coli* ribosomal proteins identified by LC-MS/MS. S2
Table S2: *S.* Typhimurium ribosomal proteins identified by LC-MS/MS. S3
Figure S1: Raw luminescence counts from a representative *in vitro* translation assay S5
Figure S2: Comparison of translation inhibition. S5
Figure S3: Concentrations of 2',3'-cA/GMP in *E. coli.* S6
Figure S4: Growth curves and fits for *E. coli*. S6

Table S1. *E. coli* ribosomal proteins identified by LC-MS/MS. Sample refers to elution conditions and nucleotide identity of the resin.

Sample	Description	Best score	# of spectra	# of unique peptides	Coverage %
500mM C	P02358 30S ribosomal protein S6	714.2	3	2	33.33
500mM C	P02359 30S ribosomal protein S7	816	10	10	48.04
500mM G	P02359 30S ribosomal protein S7	709.4	4	4	26.26
500mM A	P02359 30S ribosomal protein S7	552.5	1	1	5.59
Boil C	P02359 30S ribosomal protein S7	769.9	5	5	35.75
Boil G	P02359 30S ribosomal protein S7	401.4	1	1	8.94
Boil U	P02359 30S ribosomal protein S7	589.3	3	3	25.7
500mM G	P02413 50S ribosomal protein L15	643.2	3	3	25.69
500mM C	P02413 50S ribosomal protein L15	607.6	3	3	27.08
Boil U	P02413 50S ribosomal protein L15	523.5	2	2	18.06
Boil C	P02413 50S ribosomal protein L15	739.4	3	3	25.69
200mM A	P0A7J7 50S ribosomal protein L11	487.3	1	1	9.86
500mM G	P0A7J7 50S ribosomal protein L11	456	1	1	9.86
500mM C	P0A7J7 50S ribosomal protein L11	403.5	1	1	9.86
200mM G	P0A7J7 50S ribosomal protein L11	538.4	1	1	9.86
500mM G	P0A7K2 50S ribosomal protein L7/L12	702.4	3	3	36.36
200mM A	P0A7K2 50S ribosomal protein L7/L12	684.7	2	2	29.75
200mM G	P0A7K2 50S ribosomal protein L7/L12	655	4	4	36.36
200mM U	P0A7K2 50S ribosomal protein L7/L12	641.4	2	2	29.75
200mM C	P0A7K2 50S ribosomal protein L7/L12	553.9	2	2	29.75
200mM G	P0A7L8 50S ribosomal protein L27	484.7	1	1	16.47
200mM C	P0A7L8 50S ribosomal protein L27	463.8	1	1	16.47
500mM C	P0A7L8 50S ribosomal protein L27	563.1	1	1	16.47
500mM C	P0A7M2 50S ribosomal protein L28	811.7	1	1	12.82
500mM G	P0A7M2 50S ribosomal protein L28	776.4	1	1	12.82
500mM A	P0A7M2 50S ribosomal protein L28	720.6	1	1	12.82
500mM U	P0A7M2 50S ribosomal protein L28	633.9	1	1	12.82
200mM C	P0A7M6 50S ribosomal protein L29	518.2	1	1	23.81
200mM A	P0A7M6 50S ribosomal protein L29	512.7	1	1	23.81
200mM G	P0A7M6 50S ribosomal protein L29	333.3	1	1	23.81
200mM U	P0A7M6 50S ribosomal protein L29	143.9	1	1	23.81
500mM G	P0A7M6 50S ribosomal protein L29	523.9	1	1	23.81
500mM C	P0A7M6 50S ribosomal protein L29	581.1	3	3	49.21
Boil C	P0A7M9 50S ribosomal protein L31	540.5	1	1	20
Boil G	P0A7M9 50S ribosomal protein L31	423.5	1	1	20
Boil U	P0A7M9 50S ribosomal protein L31	353.8	1	1	20
500mM G	P0A7N4 50S ribosomal protein L32	319.7	1	1	26.32
500mM G	P0A7N9 50S ribosomal protein L33	566.4	2	2	27.27
500mM U	P0A7N9 50S ribosomal protein L33	462	1	1	27.27
500mM C	P0A7N9 50S ribosomal protein L33	456.8	2	2	30.91
500mM A	P0A7N9 50S ribosomal protein L33	423.2	1	1	27.27
500mM G	P0A7P5 50S ribosomal protein L34	398.4	1	1	17.39
500mM C	P0A7R1 50S ribosomal protein L9	829.1	8	8	67.11
500mM G	P0A7R1 50S ribosomal protein L9	756.1	7	7	57.72
500mM A	P0A7R1 50S ribosomal protein L9	435	1	1	5.37
Boil C	P0A7S3 30S ribosomal protein S12	418	3	2	21.77
200mM G	P0A7S3 30S ribosomal protein S12	275.3	1	1	13.71
500mM C	P0A7S3 30S ribosomal protein S12	395.3	1	1	21.77

Boil G	P0A7S3 30S ribosomal protein S12	138.5	1	1	13.71
Boil U	P0A7S3 30S ribosomal protein S12	364.9	1	1	21.77
500mM G	P0A7S9 30S ribosomal protein S13	850.8	4	4	46.61
500mM G	P0A7T3 30S ribosomal protein S16	460.2	3	3	42.68
500mM G	P0A7T7 30S ribosomal protein S18	719	3	3	37.33
500mM G	P0A7U3 30S ribosomal protein S19	769.3	3	3	28.26
500mM C	P0A7V0 30S ribosomal protein S2	635	2	2	14.94
Boil U	P0A7V3 30S ribosomal protein S3	806.4	4	4	30.47
500mM C	P0A7V8 30S ribosomal protein S4	760.7	8	8	43.69
500mMC	P0A7W1 30S ribosomal protein S5	613	3	3	26.95
500mM G	P0A7W7 30S ribosomal protein S8	774.6	2	2	20
500mM G	P0A7X3 30S ribosomal protein S9	605.2	1	1	6.15
Boil C	P0AA10 50S ribosomal protein L13	737.8	8	7	52.11
500mM G	P0ADY7 50S ribosomal protein L16	775.9	2	2	22.06
500mM C	P0ADZ4 30S ribosomal protein S15	495.9	5	5	42.7
500mM G	P0AG44 50S ribosomal protein L17	615.3	4	4	26.77
1M G	P0AG51 50S ribosomal protein L30	734.9	3	3	33.9
500mM C	P0AG55 50S ribosomal protein L6	559.1	1	1	6.78
500mM C	P0AG59 30S ribosomal protein S14	544.7	2	2	28.71
200mM U	P0AG63 30S ribosomal protein S17	447.7	1	1	9.52
500mM C	P0AG67 30S ribosomal protein S1	887	15	15	37.16
200mM A	P0C018 50S ribosomal protein L18	699	1	1	19.66
Boil C	P60422 50S ribosomal protein L2	645.5	7	7	34.8
500mM G	P60723 50S ribosomal protein L4	671.2	1	1	7.46
200mM G	P61175 50S ribosomal protein L22	501.1	3	3	33.64
Boil C	P62399 50S ribosomal protein L5	699.2	8	8	49.16
1M G	P68679 30S ribosomal protein S21	572.5	2	2	16.9
500mM C	P68919 50S ribosomal protein L25	559.2	2	2	25.53
500mM C	P02358 30S ribosomal protein S6	714.2	3	2	33.33

Table S2. *S.* Typhimurium ribosomal proteins identified by LC-MS/MS. Sample refers to nucleotide identity of the resin and elution conditions.

Sample	Accession	Description	Score	Coverage	# Unique Peptides	# Peptides
A1M	A0A0F6AWG8	30S ribosomal protein S20	13.03	40.23	4	4
C1M	A0A0F6AWG8	30S ribosomal protein S20	9.93	40.23	3	3
A1M	A0A0F6AX20	30S ribosomal protein S2	8.33	9.96	2	2
C1M	A0A0F6AX20	30S ribosomal protein S2	5.21	12.03	2	2
A1M	A0A0F6AZD0	30S ribosomal protein S1	11.72	11.49	5	5
A200mM	A0A0F6B3V1	50S ribosomal protein L25	29.24	69.15	4	4
A500mM	A0A0F6B3V1	50S ribosomal protein L25	10.66	35.11	3	3
C500mM	A0A0F6B3V1	50S ribosomal protein L25	13.67	26.60	3	3
A1M	A0A0F6B5A6	50S ribosomal protein L19	3.72	13.91	2	2
A500mM	A0A0F6B768	30S ribosomal protein S15	4.76	8.99	2	2
A1M	A0A0F6B790	50S ribosomal protein L21	6.45	21.36	2	2
A1M	A0A0F6B7D5	30S ribosomal protein S9	4.84	15.38	2	2
A1M	A0A0F6B7L0	50S ribosomal protein L17	5.75	26.77	3	3
C1M	A0A0F6B7L0	50S ribosomal protein L17	6.86	20.47	3	3
A1M	A0A0F6B7L2	30S ribosomal protein S4	9.91	20.87	4	4
A1M	A0A0F6B7L3	30S ribosomal protein S11	3.93	15.50	2	2
A1M	A0A0F6B7L4	30S ribosomal protein S13	6.96	26.27	3	3

A1M	A0A0F6B7L7	50S ribosomal protein L15	13.18	41.67	5	5
A1M	A0A0F6B7L9	30S ribosomal protein S5	4.48	18.56	2	2
A1M	A0A0F6B7M1	50S ribosomal protein L6	4.76	14.69	2	2
C boil	A0A0F6B7M1	50S ribosomal protein L6	8.21	14.69	2	2
A1M	A0A0F6B7M4	50S ribosomal protein L5	4.14	10.61	2	2
C1M	A0A0F6B7M4	50S ribosomal protein L5	11.87	31.84	5	5
A1M	A0A0F6B7M5	50S ribosomal protein L24	9.47	37.50	4	4
A500mM	A0A0F6B7M7	30S ribosomal protein S17	4.76	20.24	2	2
C500mM	A0A0F6B7M7	30S ribosomal protein S17	10.12	41.67	3	3
A1M	A0A0F6B7N1	50S ribosomal protein L22	4.57	19.09	2	2
A1M	A0A0F6B7N5	50S ribosomal protein L4	5.45	11.94	2	2
A1M	A0A0F6B7N6	50S ribosomal protein L3	18.84	41.63	7	7
A boil	A0A0F6B7N6	50S ribosomal protein L3	14.71	12.92	3	3
A1M	A0A0F6B7N7	30S ribosomal protein S10	7.19	24.27	3	3
A boil	A0A0F6B7N7	30S ribosomal protein S10	30.58	29.13	3	3
A1M	A0A0F6B7P4	30S ribosomal protein S7	22.98	41.67	7	7
A1M	A0A0F6B7P5	30S ribosomal protein S12	7.42	17.74	3	3
C500mM	A0A0F6B909	50S ribosomal protein L34	4.61	19.57	2	2
C200mM	A0A0F6B909	50S ribosomal protein L34	4.16	19.57	2	2
A500mM	A0A0F6B9S9	50S ribosomal protein L31	8.83	40.00	2	2
C500mM	A0A0F6B9S9	50S ribosomal protein L31	21.95	74.29	4	4
A1M	A0A0F6B9Y0	50S ribosomal protein L11	12.54	16.90	2	2
A boil	A0A0F6B9Y0	50S ribosomal protein L11	38.82	16.90	2	2
A1M	A0A0F6B9Y1	50S ribosomal protein L1	30.53	40.60	7	7
A boil	A0A0F6B9Y1	50S ribosomal protein L1	93.56	41.45	7	7
A1M	A0A0F6B9Y2	50S ribosomal protein L10	11.07	31.52	4	4
A boil	A0A0F6B9Y2	50S ribosomal protein L10	63.09	31.52	4	4
A1M	A0A0F6B9Y3	50S ribosomal protein L7/L12	18.17	28.10	4	4
A boil	A0A0F6B9Y3	50S ribosomal protein L7/L12	40.57	19.01	2	2
A1M	A0A0F6BAQ8	30S ribosomal protein S6	12.01	29.01	5	5
A1M	A0A0F6BAR0	30S ribosomal protein S18	4.48	26.67	2	2
C1M	A0A0F6BAR0	30S ribosomal protein S18	3.93	29.33	2	2
A1M	A0A0F6BAR1	50S ribosomal protein L9	33.48	51.68	8	8

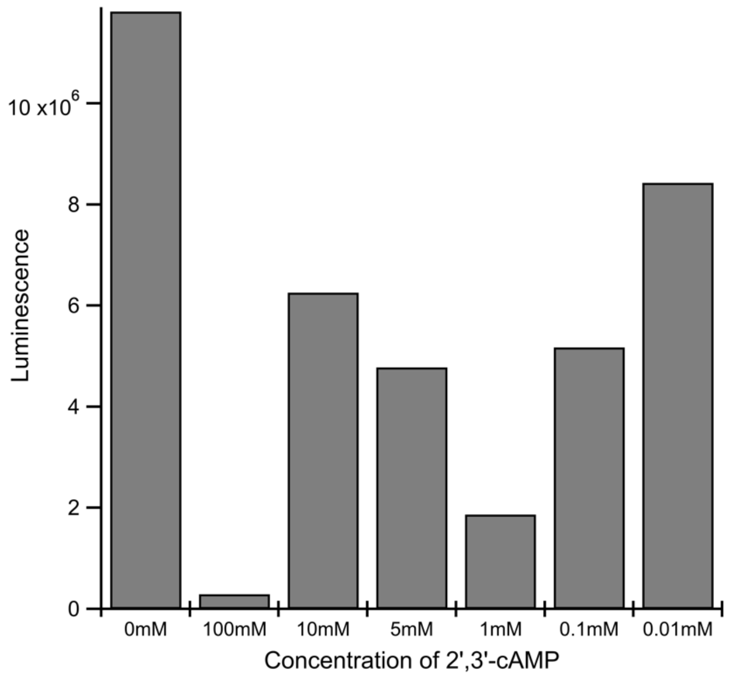

Figure S1. Raw luminescence counts from a representative *in vitro* translation assay in the presence of different concentrations of 2’,3’-cAMP. Luminescence was measured at 560 nm and generated by NanoLuc enzymatic activity.

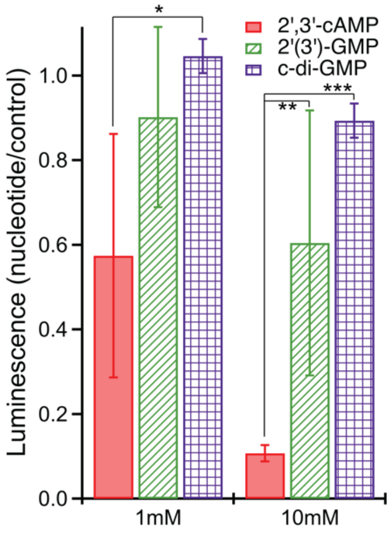

Figure S2. Comparison of translation inhibition by 2’,3’-cAMP, linear 2’(3’)-GMP, and cyclic di-GMP. *, P = 0.0257; **, P = 0.0401; ***, P = 0.0011. Differences in inhibition at 1 mM vs. 10 mM were not significant for 2’/3’-AMP or c-di-GMP.

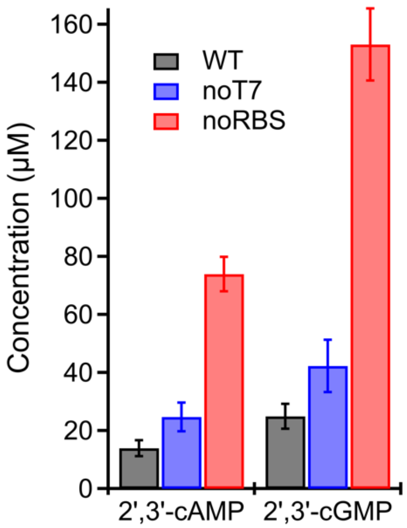

Figure S3. Concentrations of 2',3'-cA/GMP in *E. coli* Tuner WT, WT with pNoT7 plasmid, and WT with pNoRBS plasmid.

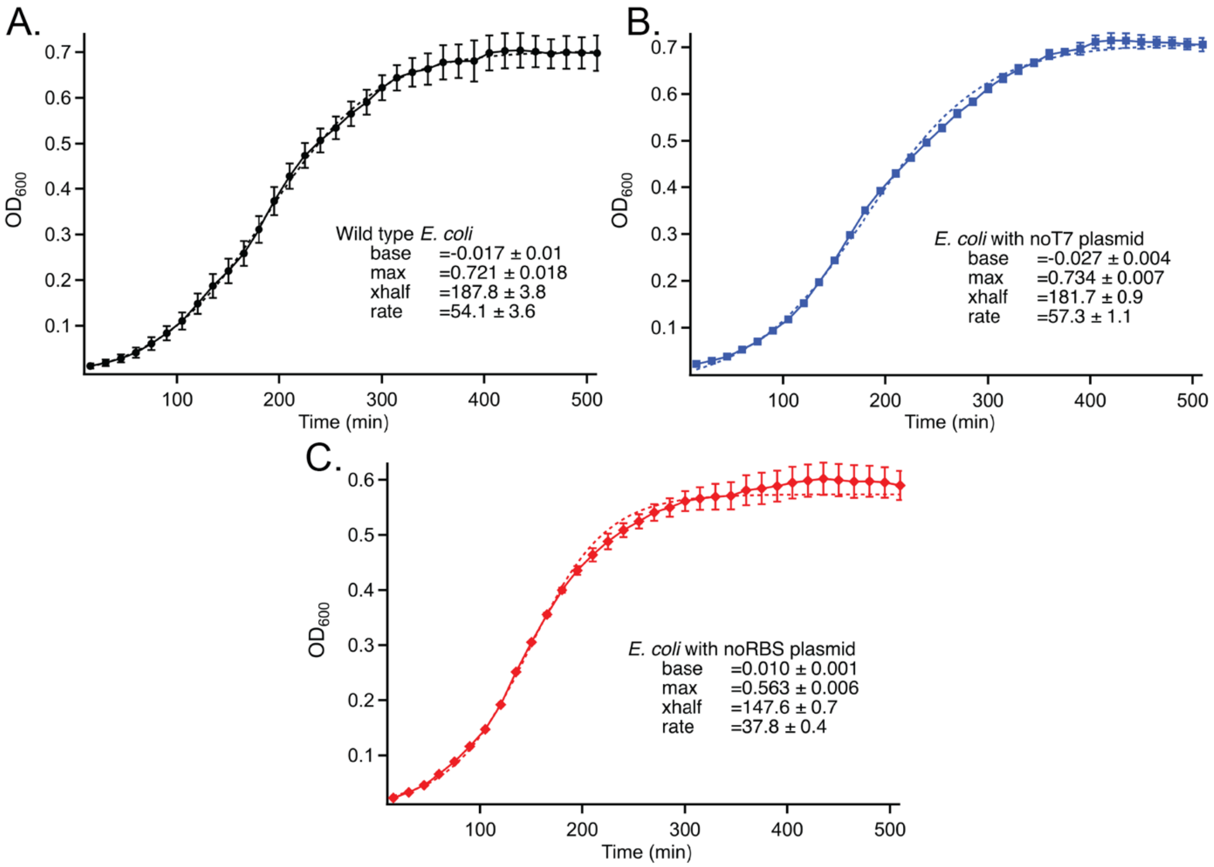

Figure S4. Growth curves and fits for *E. coli* WT, WT with pNoT7 plasmid, and WT with pNoRBS plasmid. Error bars at each time point represent +/- standard deviation. Sigmoidal fits are shown as dashed lines and fitting parameters (+/- standard deviation) are listed in the figure legends.
